# Predicting Recovery of Cognitive Function Soon after Stroke: Differential Modeling of Logarithmic and Linear Regression

**DOI:** 10.1371/journal.pone.0053488

**Published:** 2013-01-11

**Authors:** Makoto Suzuki, Yuko Sugimura, Sumio Yamada, Yoshitsugu Omori, Masaaki Miyamoto, Jun-ichi Yamamoto

**Affiliations:** 1 Faculty of Medical Technology, Niigata University of Health and Welfare, Niigata, Japan; 2 Department of Rehabilitation Medicine, Kawasaki Municipal Tama Hospital, Kawasaki, Japan; 3 Department of Rehabilitation Science, Nagoya University Graduate School of Medicine, Nagoya, Japan; 4 Department of Rehabilitation Medicine, St. Marianna University, Yokohama City Seibu Hospital, Yokohama, Japan; 5 Department of Rehabilitation Medicine, Fuchinobe General Hospital, Sagamihara, Japan; 6 Graduate School of Human Relations, Keio University, Minato-ku, Japan; University of Pittsburgh, United States of America

## Abstract

Cognitive disorders in the acute stage of stroke are common and are important independent predictors of adverse outcome in the long term. Despite the impact of cognitive disorders on both patients and their families, it is still difficult to predict the extent or duration of cognitive impairments. The objective of the present study was, therefore, to provide data on predicting the recovery of cognitive function soon after stroke by differential modeling with logarithmic and linear regression. This study included two rounds of data collection comprising 57 stroke patients enrolled in the first round for the purpose of identifying the time course of cognitive recovery in the early-phase group data, and 43 stroke patients in the second round for the purpose of ensuring that the correlation of the early-phase group data applied to the prediction of each individual's degree of cognitive recovery. In the first round, Mini-Mental State Examination (MMSE) scores were assessed 3 times during hospitalization, and the scores were regressed on the logarithm and linear of time. In the second round, calculations of MMSE scores were made for the first two scoring times after admission to tailor the structures of logarithmic and linear regression formulae to fit an individual's degree of functional recovery. The time course of early-phase recovery for cognitive functions resembled both logarithmic and linear functions. However, MMSE scores sampled at two baseline points based on logarithmic regression modeling could estimate prediction of cognitive recovery more accurately than could linear regression modeling (logarithmic modeling, R^2^ = 0.676, *P*<0.0001; linear regression modeling, R^2^ = 0.598, *P*<0.0001). Logarithmic modeling based on MMSE scores could accurately predict the recovery of cognitive function soon after the occurrence of stroke. This logarithmic modeling with mathematical procedures is simple enough to be adopted in daily clinical practice.

## Introduction

Cognitive disorders in the acute stage of stroke are common and are important independent predictors of adverse outcome in the long term [1]. Studies have reported that up to 50% of stroke survivors experience new onset or worsening of cognitive impairment after the stroke [2]–[4]. Stroke-related cognitive deficits interfere with functional recovery and the potential benefits of rehabilitation [1]–[5]. Furthermore, the presence of cognitive impairment is known as an important predictor of recovery and has been associated with the risk of recurrent stroke [6]. Cognitive impairment resulting from stroke can have a devastating impact on both patients and their families [5], [6].

Several studies have shown a relation between cognitive impairment and the site of brain lesions [6], concomitant with white matter lesions [7]–[9], aging [10], [11], hypertension [11], and diabetes [12]. In addition, in relation to the recovery of cognitive function, the initial degree of cognitive impairment, previous stroke, and the presence of cortical atrophy at stroke onset are considered as predictors for the recovery from cognitive impairments after the stroke [13], [14]. Nys et al. [15], however, suggested that there is a very large inter-individual variation in the test interval for cognitive assessment and that the degree of cognitive recovery is greatest in the first month post stroke. Previous studies suggested that baseline functional status is a stronger predictor for the recovery of functions than are multidimensional risk factors because the initial patterns of recovery could be affected by any of a number of multiple factors [16], [17]. Longitudinal studies on stroke recovery have traditionally focused on correlational analysis using linear regression modeling [18]–[25]. Moreover, single-subject research has assessed the slope of a trend or the rate of change within the individual's data so that individual recovery could be deduced from the correlation of the group data [16], [17], [26]–[28]. However, stroke patients typically show nonlinear recovery patterns [29]. In general, cognitive and motor dysfunction and activity limitation show rapid recovery during the acute phase and reach a plateau or level off after several months following onset [15]–[17], [25]. Koyama et al. [16] and Suzuki et al. [17] examined the validity and the applicability of logarithmic modeling for predicting the functional recovery of stroke patients with hemiplegia. These studies noted that the time-course observation data plotted for each individual were closely related to the data change derived from the predictive model. However, despite the fact that prediction of cognitive recovery can give important information to both patients with cognitive disorders and their families, most of the studies have focused on functional recovery [16]–[26]. Therefore, it is still difficult to predict the extent and duration of cognitive impairments. Several questions need to be addressed, such as does the time course of early-phase recovery for cognitive function resembled a logarithmic or linear regression model, and which is a stronger predictor of recovery of cognitive functions, logarithmic or linear regression modeling? For both service providers and receivers, accurate prediction enables effective use of resources by allowing better estimation of such factors as length of hospitalization [24]. Thus, for both individual patients and health care administrators, accurate prediction of cognitive recovery would provide crucially important information.

Therefore, we conducted a longitudinal study to identify predictors of the recovery of cognitive function soon after stroke and to predict the recovery of cognitive function by differential modeling of logarithmic and linear regression. From the findings of previous studies in predicting functional recovery [16], [17], we hypothesized that (a) the time course of the early phase of cognitive recovery would resemble a logarithmic regression model, and (b) the recovery of cognitive function could be predicted accurately by a logarithmic regression model based on the slope of the early phase of cognitive recovery. The present study would be the first to show predictive value for cognitive recovery by applying logarithmic regression modeling.

## Materials and Methods

### Subjects

Two rounds of data collection were performed in the prediction of cognitive recovery: the first was for the purpose of identifying the time course of cognitive recovery in the early phase of group data. The second round of data collection was for the purpose of ensuring that the correlation of the group data applied to the prediction of each individual's degree of cognitive recovery and for predicting individual cognitive recovery by logarithmic and linear regression modeling based on the individual slope of the early phase of cognitive recovery. Eligibility criteria included stroke, ability to sit up with a backrest for more than 30 minutes, absence of aphasia, agnosia, and apraxia, absence of severe cardiorespiratory insufficiency, no history of dementia and neuromuscular disease, and the desire to participate. Sample size in the first round of data collection was based on a desired 90% statistical power to detect 0.5 effect size (r) in the Mini-Mental State Examination (MMSE) score, with a two-sided α of 1%. A sample size of 52 was derived by insertion of 1-power (0.90), α (0.01), and effect size (0.50) values in the Hulley matrix [30]. We adopted stricter sample size estimation in the second round of data collection for accurate prediction: a desired 95% statistical power to detect 0.6 effect size (r) in the MMSE score with a two-sided α of 1%. A sample size of 40 was derived by insertion of 1-power (0.95), α (0.01), and effect size (0.60) values in the Hulley matrix [30]. The authors therefore planned to recruit approximately 50 and 40 patients for the first and second rounds of data collection, respectively, in this study. All patients received standard stroke treatments and physical or occupational therapies. The study was approved by the Kawasaki Municipal Tama Hospital Institutional Committee on Human Research. All subjects and their families were briefed about the aims of the study and the testing procedure prior to participation. Written informed consent was obtained from each subject and/or their family. This study was performed in accordance with the Declaration of Helsinki.

### Assessment of cognitive impairment

The MMSE is widely used for the assessment of cognitive mental status in both clinical practice and research [31]. This instrument was originally developed to screen for dementia and delirium in a psychiatric setting and has been shown to have good reliability, sensitivity, and specificity [32]. Many studies now use it as a screening instrument for “global cognitive impairment” [33]. It assesses the subject's orientation, attention, immediate and short-term recall, language, and ability to follow simple verbal and written instruction. MMSE scores can range from 0 to 30, and lower scores indicate greater cognitive impairment [32]. The MMSE has test-retest stability, classification accuracy, and construct and criterion-related validity for stroke patients [34].

### Procedure

To identify the time course of cognitive recovery in the early phase after stroke, the MMSE assessments were carried out on three occasions for the first round of data collection: initial assessment from onset of stroke (baseline assessment) and then at 1 week (second set of assessments) and 2 weeks (third set of assessments) after the baseline assessment. In addition, to ensure that the correlation of the group data in the early phase applied to the prediction of each individual's degree of cognitive recovery, the MMSE assessments were carried out on four occasions for the second round of data collection: baseline assessment and at 1, 2, and 3 weeks after the baseline assessment in each individual.

### Data analysis

Friedman's test was performed to compare time course differences in MMSE scores. For post hoc analysis, differences in MMSE scores were analyzed by the Wilcoxon signed-rank test. Conventional logarithmic and linear regression analyses were performed to identify the time course of cognitive recovery in the early phase. The MMSE scores were regressed on the logarithm and linear of time given by *f (t) = a+b ln (t)* and *f (t) = a+b (t)*, where *t* is the number of days since stroke onset, *a* is the MMSE score at stroke onset, and *b* is the slope of early-phase cognitive recovery. To assess the fit of the predictive model, we tested the fit of the time course of cognitive recovery and used conventional logarithmic and linear regression models according to the coefficient of determination (R^2^).

**Figure 1:**
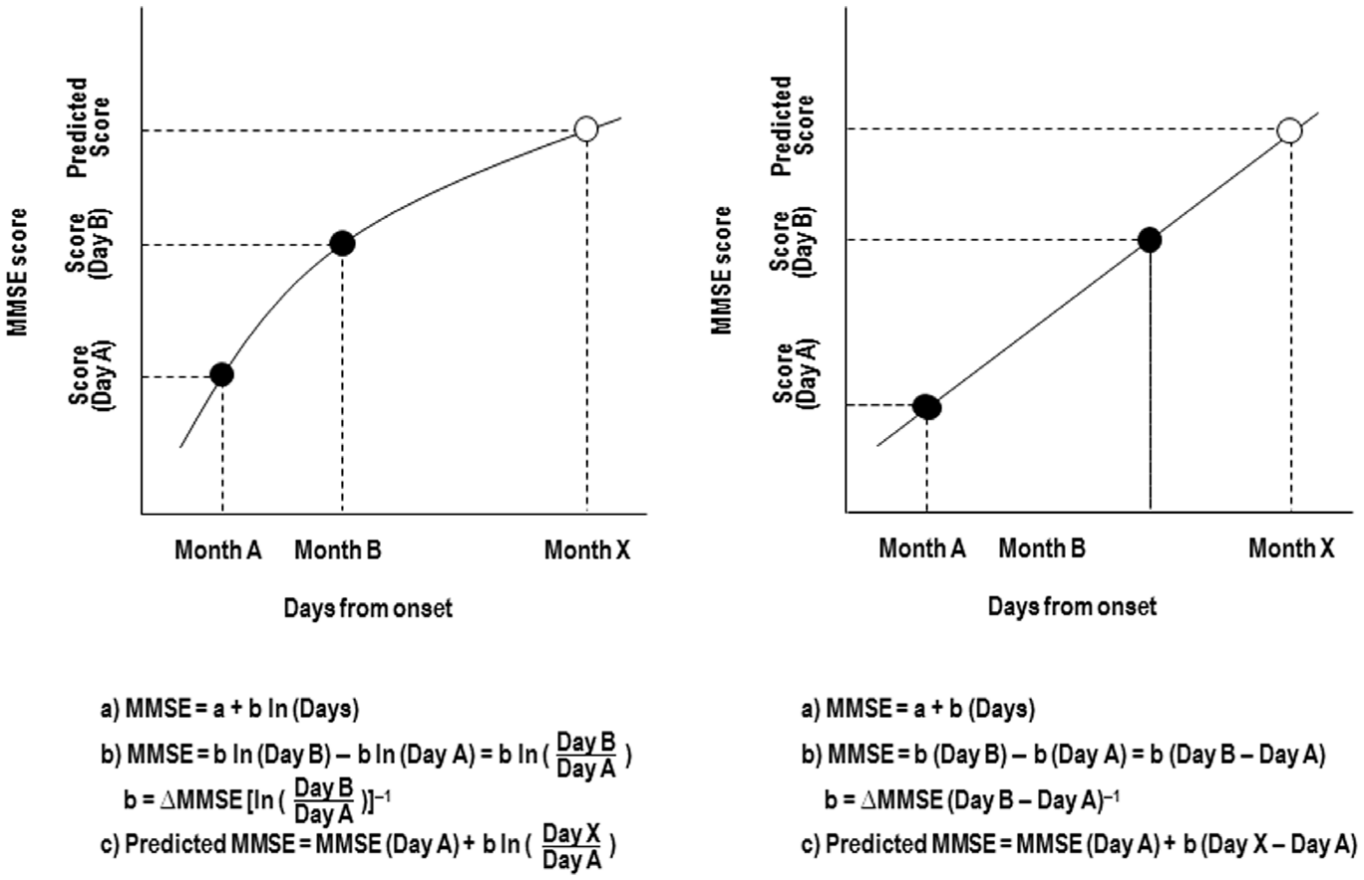
Logarithmic modeling and linear regression modeling. A generic structure of logarithmic (A) and linear regression (B) modeling is given in a simple formula (independent variable = days from onset). MMSE: Mini-Mental State Examination; Ln: natural logarithm. ΔMMSE indicates change in MMSE scores between Day A and Day B. X can be calculated with this formula.

In addition, in order that the correlation of the group data in the early phase applied to the prediction of each individual's degree of cognitive recovery, we performed calculations on the MMSE score at the first two time-points after admission (baseline and second set of assessments with the MMSE). For each patient, the increase in MMSE score between these two time-points (Δ MMSE) was used as the basis for scaling coefficient (*b*) in the equation. Thus, using the scores at the initial two sampling points, these equations could be tailored to forecast each patient's cognitive recovery (model formula in Figure 1) [16], [17]. To assess the applicability of the predictive model on an individual basis, a conventional linear regression analysis was performed to compare the MMSE score that was actually obtained (from the third and fourth sets of assessment) with the predicted values that were derived from the model formula. To assess the individual applicability of logarithmic and linear modeling, a conventional linear regression analysis was performed to compare the MMSE score that was actually obtained (from the third and fourth sets of assessment) with the predicted values that were derived from the model formula [16], [17]. For this analysis, we excluded the scores obtained at the first two sampling points (denoted by filled symbols in Figure 1).

Molly et al. [35] demonstrated that mean distribution of test-retest differences in MMSE lay within 2.4 points. Therefore, when the difference between actual and predicted values was 3 points or less, the predicted values were considered to be correct. Subjects were classified into two groups: those with MMSE scores of 3 points or less difference between actual and predicted values and those with more than 3 points difference. Differences in categorical variables (correct and incorrect) were analyzed with the χ2 test. A *P* value of <0.05 was considered statistically significant. All statistical procedures were carried out with PASW Statistics 18 software (IBM, New York, USA).

## Results

### Profile of recovery of cognitive impairment

**Figure 2:**
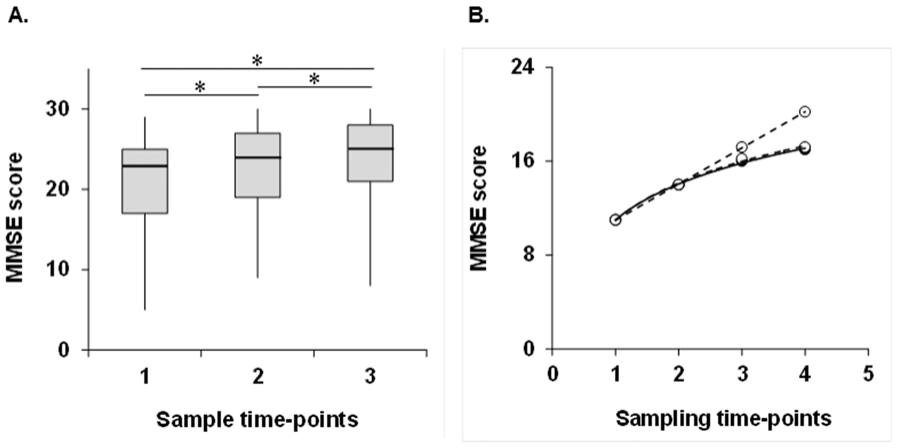
Time course of MMSE score. Plots of actual MMSE scores (filled symbols) from early-phase group data (A) and actual (filled symbols) and predicted MMSE scores (open symbols) from a representative subject (B) are shown. In A, the middle line in the box: median; the ends of the box: interquartile range of the median; the bars: ranges of data distribution; asterisk: *P*<0.0001 in the Wilcoxon signed-rank test. The MMSE score increased significantly over the 3 sets of assessment (Friedman's test, *P*<0.0001). In B, the pattern of increase in the predicted values that were derived from the logarithmic model formula was similar to the pattern from the MMSE scores of the representative subject that were actually obtained. However, linear regression modeling overestimated the prediction of cognitive recovery to a greater degree compared with the logarithmic approach. MMSE: Mini-Mental State Examination.

In the first round of data collection, between April 17, 2006, and November 12, 2008, 57 patients (25 men, 32 women, mean age 73.5±9.3 [SD] years) were enrolled from the participating hospitals. Characteristics of the patients are presented in Table 1. There were 47 patients with cerebral infarction and 10 with cerebral hemorrhage. The test patients included 14 participants with a classification of partial anterior circulation infarct (PACI), 15 with a classification of posterior circulation infarct (POCI), and 18 with a classification of lacunar infarcts (LACI) according to the Oxfordshire stroke classification [36]. There were 4 patients with thalamus hemorrhage, 1 with putamen hemorrhage, 1 with cerebellum hemorrhage, 4 with hemorrhage at other sites. Leuko-araiosis scores [9] for the 57 patients ranged from 0 to 37 points (median, 10 points; interquartile range [IQR], 6–13 points). Baseline MMSE scores for the 57 patients ranged from 5 to 29 points (median, 23 points; IQR, 17–25 points). The average time since the stroke event was 9.3±8.1 days. Time-series plots of early-phase MMSE scores for all 57 subjects are shown in Figure 2A. The MMSE score increased significantly over the 3 sets of assessment (Friedman's test, *P*<0.0001; Fig. 2A). The Wilcoxon signed-rank test showed that the MMSE score significantly increased in comparison with the baseline MMSE score. Although both logarithmic and linear regression modeling had high coefficient of determination (R^2^) values, the R^2^ value of logarithmic regression modeling was slightly higher than that of linear regression modeling (logarithmic modeling, R^2^ = 0.95, *P*<0.0001; linear modeling, R^2^ = 0.94, *P*<0.0001).

**Table 1:** Baseline Characteristics of the Study Group.

Characteristic	First round	Second round
Participants (n)	57	43
Age (y)	73.5±9.3	72.4±10.8
Sex (n)		
Male	25	19
Female	32	24
Diagnosis (n)		
Infarction	47	36
PACI	14	10
POCI	15	9
LACI	18	17
Hemorrhage	10	7
Thalamus	4	2
Putamen	1	0
Cerebellum	1	1
Other	4	4
Leuko-araiosis score	10 (6–13)	9 (4–12)
Days post-stroke at assessment	9.3±8.1	8.1±8.7
Mini-Mental State Examination	23 (17–25)	23 (20–25)

Values are mean ± SD, n, or median (interquartile range).

PACI, partial anterior circulation infarct; POCI, posterior circulation infarct; LACI, lacunar infarcts.

### Assessment of model fit

In the second round of data collection, we assessed the rate of change (*b*) within each individual's early-phase data so that the individual's recovery could be deduced from the correlation of the group data. Between September 19, 2006, and February 13, 2012, 43 stroke patients satisfied eligibility criteria (Table 1). There were 36 patients with cerebral infarction and 7 with cerebral hemorrhage. The test patients included 10 participants with a classification of PACI, 9 with a classification of POCI, and 17 with a classification of LACI. There were 2 patients with thalamus hemorrhage, 1 with cerebellum hemorrhage, 4 with hemorrhage at other sites. Leuko-araiosis scores [9] for the 43 patients ranged from 0 to 33 points (median, 9 points; interquartile range [IQR], 4–12 points). Baseline MMSE scores for the 43 patients ranged from 11 to 29 points (median, 23 points; IQR, 20–25 points). The average time since the stroke event was 8.1±8.7 days.

Two baseline MMSE scores were sampled and are denoted by filled symbols in Figure 1. Logarithmic and linear model formulae could be tailored to forecast each patient's functional recovery using sampled baseline MMSE scores. Time-series plots of MMSE data for a representative subject are shown in Figure 2B. For this subject, MMSE scores have been regressed on the logarithm and linear of time. The pattern of increase in the predicted values that were derived from the logarithmic model formula was similar to the MMSE scores that were actually obtained, confirming the predictability of cognitive recovery. In contrast, linear regression modeling overestimated the predicted cognitive recovery to a greater degree compared with the logarithmic approach.

**Figure 3:**
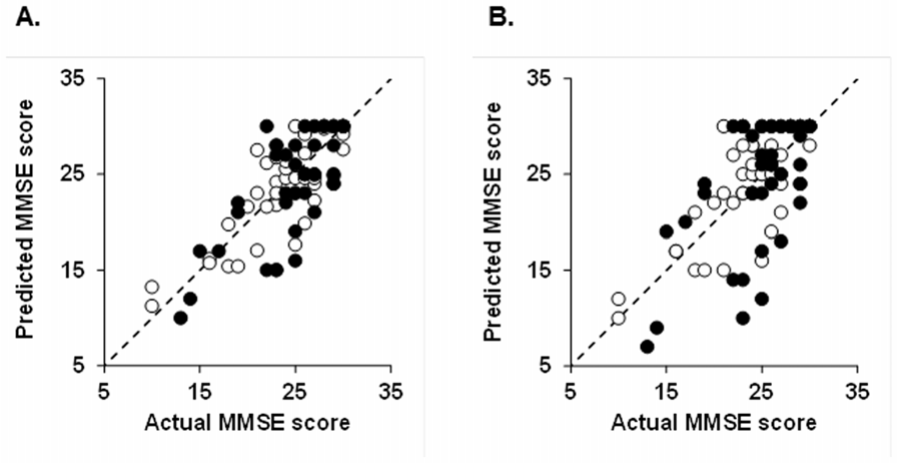
Scatterplots showing the relations between MMSE scores actually obtained and predicted MMSE scores. Predicted and actual MMSE scores at the third (open symbols) and fourth (filled symbols) sets of assessment by logarithmic model (A) and linear regression model (B). Logarithmic regression modeling estimated prediction of cognitive recovery to a more accurate degree than did the linear approach (logarithmic modeling, third set of assessments: R^2^ = 0.676, *P*<0.0001, fourth set of assessments: R^2^ = 0.521, *P*<0.0001; linear regression modeling, third set of assessments: R^2^ = 0.598, *P*<0.0001, fourth set of assessments: R^2^ = 0.370, *P*<0.0001).

Regression analysis was conducted to compare actual data and predicted values and thus to determine whether the modeling formulae accurately predicted the obtained MMSE scores. The R^2^ value between the actual and predicted MMSE scores for the third set of assessments was high for logarithmic regression modeling (R^2^ = 0.676, *P*<0.0001) but moderate (R^2^ = 0.598, *P*<0.0001) for linear regression modeling (Table 2 and Figure 3). The R^2^ values for the fourth set of assessments were moderate for both logarithmic (R^2^ = 0.521, *P*<0.0001) and linear (R^2^ = 0.370, *P*<0.0001) regression modeling. However, the R^2^ value of logarithmic modeling based on baseline MMSE scores was higher than that of linear regression modeling based on baseline MMSE scores for predicting the third and fourth sets of MMSE scores (Table 2 and Figure 3). For the third set of assessments, there were 33 (76.7%) patients with MMSE scores of 3 points or less difference between actual and predicted values by the logarithmic model, whereas there were 30 (70.0%) patients by the linear model (*P*<0.0001; Table 2). For the fourth set of assessments, there were 29 (67.47%) patients with MMSE scores of 3 points or less difference between actual and predicted values by the logarithmic model, whereas there were 23 (53.5%) patients by the linear model (*P*<0.0001; Table 2).

**Table 2:** Profile of Recovery on Mini-Mental State Examination.

	Third set of assessments	Fourth set of assessments
**Actual MMSE score**	**24 (22–27)**	**25 (23–28)**
Predicted MMSE score		
Logarithmic model	25 (22–27)	25 (22–28)
Linear regression model	25 (22–28)	26 (23–30)
Model fit, R^2^		
Logarithmic model	0.68*	0.52*
Linear regression model	0.60*	0.37*
Difference between actual and predicted MMSE score		
Logarithmic model	0.0±2.9	−0.8±3.8
Linear regression model	0.3±3.5	−0.5±5.2
%Patients within 3 point difference		
Logarithmic model	72.1†	67.4†
Linear regression model	70.0	53.5

Values are mean ± SD or median (interquartile range).

MMSE: Mini-Mental State Examination.

* *P*<0.0001 for difference between actual and predicted values (linear regression analysis).

† *P*<0.0001 for difference between logarithmic and linear regression model (χ2 test).

## Discussion

In this study, the recovery of cognitive function soon after stroke was predicted. Our results indicated that (a) the time course of early-phase recovery for cognitive functions resembled both logarithmic and linear functions for group data, whereas (b) MMSE scores which sampled at two points based on logarithmic regression modeling could be used to predict accurate patterns of individual recovery of cognitive function. Especially, prediction based on linear regression modeling overestimated cognitive recovery when compared with prediction based on logarithmic modeling. Moreover, the differences between actual and predicted values for logarithmic regression modeling were lower than those for linear regression modeling. Thus, the model formula based on the logarithmic function could be a useful tool for predicting cognitive recovery of stroke patients with cognitive impairment.

Koyama et al. [16] and Suzuki et al. [17] showed that the predicted values using logarithmic modeling enabled powerful and accurate forecasting of functional recovery. However, Koyama et al. [16] suggested that the model might not be applicable for patients whose clinical manifestations are mainly cognitive rather than motor. Along with this knowledge provided by the Koyama et al. report [16], we developed a predictive model that was exclusive to cognitive impairment soon after the stroke. An additional new observation in the present study was that logarithmic modeling could accurately predict recovery of cognitive function as well as that of motor function soon after the stroke. Moreover, logarithmic modeling was the stronger predictor for the recovery of cognitive function than was linear regression modeling. Stroke-related cognitive deficits interfere with functional recovery and the potential benefits of rehabilitation [1], [5] and are an important predictor of recovery [6]. In the present study, we used paired data from the baseline and the second MMSE samplings after the onset of the stroke to maintain consistency. Any pair was suitable for defining the coefficient (*b*) of the model formula. The flexibility of the model formula enabled easy re-estimation if predictive and actual values deviated. The simplicity and the flexibility of using the logarithmic model formula could make it the better fit for clinical applications.

For representative subjects, the predicted values that were derived from the logarithmic modeling were similar to the actual MMSE scores, whereas those values derived from linear regression modeling overestimated the actual scores. Some authors have reported an overall improvement in cognitive function [15], [37], whilst others have reported a decline [38], [39]. This implies that linear modeling might greatly overestimate the prediction of cognitive recovery in comparison with that by logarithmic modeling. There was, however, no clear understanding of the effect of many symptoms and their relation to cognitive decline. Therefore, a larger number of participants would be needed in further studies to investigate cognitive decline in relation to lesion types (e.g., site of the lesion, white matter lesions, and brain atrophy) and other attributes, such as patient age, sex, and comorbidities. In addition, about 20% of patients who had cognitive deficits 1 month after the stroke were reported to show normal cognitive functioning at 6 months after stroke [40], and 10% of the stroke patients with cognitive impairment had recovered after a year [14]. Despite these improvements, most stroke patients show no improvement or even a decline in cognitive function [41]. Cognitive impairment after stroke was common, occurring in 17% of 1-year survivors [42]. In the present study, we found that logarithmic modeling might be useful even at earlier stages of illness and during shorter periods of hospitalization, but its ability to predict recovery of cognitive function over the longer term after the stroke remains unclear, where its refers to logarithmic modeling. Therefore, further studies will be needed to determine the applicability of logarithmic modeling to MMSE data collected in the later phases of the condition.

Prior studies have pointed out the limitations of assessment when using the MMSE to assess frontal lobe functions such as executive skills and right-hemisphere functions, visuospatial and constructional skills, and also known “floor” and “ceiling” effects [6], [15]. In the present study, eligibility criteria included absence of aphasia, agnosia, and apraxia, and individuals who did not meet these criteria were excluded from the study. These countermeasures minimized evaluation bias when using the MMSE. In contrast, strict sampling criteria may inhibit the generalization of prediction based on logarithmic modeling. Although the MMSE is a core component of cognitive assessment worldwide and continues to be used in clinical research, the Modified Mini-Mental State Examination (3MSE) has been developed to extend the ceiling and floor of the test, to sample a wider range of cognitive abilities, and to enhance the reliability and validity of the scores [43]. It is thus necessary to investigate the relation between recovery and decline of cognitive function and the results of detailed tests using the 3MSE to assess various cognitive impairments including frontal lobe function and right-hemisphere functions.

This study did not examine covariates [6]–[9], [13]. Tatemichi et al. [13] conducted a prospective cohort study to develop a predictive model for the incidence of cognitive impairments using information about potential risk factors after stroke. In their study, when the lesions were classified by sites, cognitive impairment was more frequent among patients with occipital, temporo-occipital, and temporoparietal lobe infarctions than among those with infarcts confined to the basal ganglia and capsule or brainstem and cerebellum. Sonohara et al. [7] investigated the relation of white matter lesions with global cognitive function. Their multiple logistic analyses revealed that the size of white matter lesions remained a significant determinant of cognitive impairment. In contrast, Tham et al. [14] examined the prevalence and natural history of cognitive impairment in a cohort of post-stroke patients. They found that patients with cognitive impairments differed significantly in age, years of education, and baseline MMSE scores compared with cognitively intact patients. The problem of defining predictors of cognitive recovery is complex as a result of the multidimensional factors caused by conditions such as site of cerebral infarction, size of white matter lesions, cerebral atrophy, age, and years of education. Some previous studies suggested the baseline function is a stronger predictor for the recovery of function than are multiple covariates [16], [17], [44]–[46]. In future studies, a larger number of participants will be needed to investigate the relation between recovery of cognitive function and multiple important covariates. With the addition of detailed examination classifying participants by their covariates and the inclusion of a large number of patients, the results of our study might be more generally applicable.

The results of our study are relevant for clinical practice because they may enhance therapy. Indeed, there are a number of different approaches that could be used in the rehabilitation of cognitive dysfunction, for example, restorative therapies, compensation or strategy training, and behavioral approaches [41]. In the future, it will be possible to select more appropriate treatment regimens as knowledge relating to recovery increases. Further research is needed to investigate the relation between the MMSE score and the effect of training and to then compare sensitivities of the MMSE score with clinical change to obtain more accurate and quantitative information on the recovery of cognitive function after stroke.

In conclusion, cognitive disorders in the acute stage of stroke interfere with the potential benefits of rehabilitation and affect both patients and their families. The quality and efficiency of rehabilitation services are improved by accurate predictions based on a proper definition of intervention goals for individual patients with cognitive impairment. Logarithmic modeling based on MMSE scores could accurately predict the recovery of cognitive function soon after stroke. This logarithmic modeling with simple mathematical procedures is suitable for daily clinical practice.
